# Screening utility and acceptability of the Kiswahili-pGALS (paediatric Gait, Arms, Legs, Spine) at a tertiary referral hospital in Kenya-A diagnostic accuracy study

**DOI:** 10.1186/s12969-023-00882-z

**Published:** 2023-09-18

**Authors:** Jacqueline E. Kawishe, Anthony Ngugi, Stanley Luchters, Helen Foster, Angela Migowa

**Affiliations:** 1 Department of Paediatrics, The Aga Khan University, Nairobi, Kenya; 2 Department of Population Health, The Aga Khan University, Nairobi, Kenya; 3 Department of International Public Health, Liverpool School of Tropical Medicine (LSTM, Liverpool, UK; 4 Centre for Sexual Health and HIV/AIDS Research (CeSHHAR), Harare, Zimbabwe; 5 Department of Public Health and Primary Care, Ghent University, Ghent, Belgium; 6 Newcastle University, Newcastle, UK; 7 Department of Paediatrics, The Aga Khan University Medical College East Africa, 30270-00100 3rd Parklands Avenue Nairobi, Nairobi, Kenya

**Keywords:** pGALS, Kenya, Kiswahili, Children, Musculoskeletal, Clinical assessment, Education

## Abstract

**Background:**

Paediatric rheumatic diseases cause considerable disease burden to children and their families (Moorthy LN, Peterson MGE, Hassett AL, et al, Pediatric Rheumatology 8:20, 2010). Delayed diagnosis is a significant determinant of severity and mortality attributed to these conditions (Foster HE, Eltringham MS, Kay LJ, et al, Arthritis Care Res 57(6):921-7, 2007). pGALS is a simple clinical tool used to assess joints and identify musculoskeletal (MSK) conditions in school-going children to enable early referral to paediatric rheumatologists.

**Objectives:**

This study aimed to translate and determine the diagnostic accuracy and acceptability of a Kiswahili version of the pGALS screening tool among Kiswahili-speaking children.

**Methods:**

The pGALS screening questions were translated into Kiswahili according to the World Health Organisation (WHO) standard for translation of a tool. The validity of the Kiswahili PGALS was ascertained and acceptability rated (time taken, discomfort). Using systematic random sampling, we enrolled children aged 5–16 years presenting at the Aga Khan University Hospital’s (AKUH) emergency department in Kenya, who spoke Kiswahili and had symptoms suggestive of an MSK condition. Those already under follow-up at the paediatric rheumatology service at AKUH were excluded. MSK assessment was undertaken by two resident doctors using the newly translated Kiswahili-pGALS and findings were compared with a paediatric rheumatologist examination (‘gold-standard’) on the same day, and who was blinded to the pGALS findings. We analysed demographic details of the participants and determined the diagnostic accuracy by cross tabulation of the index test results by the results of the reference standard.

**Results:**

One hundred children with a median age of nine years (IQR 7–11) were enrolled. The sensitivity and specificity of the Kiswahili-pGALS screening tool were 76.8% (95%CI 63.6–87.0%) and 40.0% (95%CI 23.9–57.9%), respectively. The diagnostic accuracy was 62.7% (95%CI 52.1–72.1%), area under the ROC was 0.58 (95%CI 0.48–0.68). The median time to perform the Kiswahili-pGALS was 5.0 min (IQR 3.5–6.0 min). Ninety percent of the guardians found the practice of Kiswahili-pGALS to have none, or only some discomfort.

**Conclusions:**

The Kiswahili-pGALS’s was found to be a useful screening tool to aid early identification of MSK conditions in Kiswahili-speaking settings. However, the low specificity implies that relatively large number of false positives would still need to be reviewed by a rheumatologist if the tool is adapted for use.

## Introduction

Paediatric rheumatic diseases are chronic illnesses that can result in disability and morbidity with considerable impact on children and their families [1]. Rheumatic diseases represent a multitude of degenerative, inflammatory, and autoimmune conditions [2]. Patients with these conditions may potentially experience severe chronic pain, joint damage, increasing disability, and even death [2]. Rheumatic conditions can be classified into inflammatory and non-inflammatory conditions [3]. Inflammatory rheumatic conditions include juvenile idiopathic arthritis (JIA), systemic lupus erythematosus (SLE), juvenile dermatomyositis (JDM), scleroderma, vasculitis, and the auto-inflammatory (recurrent fever) syndromes among others [3]. Non-inflammatory rheumatic conditions include mechanical conditions (mostly orthopaedic), the amplified musculoskeletal pain syndromes, and hereditary syndromes [3].

Delayed presentation to a paediatric rheumatologist is a significant determinant of adverse clinical outcomes attributed to rheumatic conditions [4]. The median time to initiate contact with a paediatric rheumatologist in patients with MSK symptoms has been estimated at 2–5 months in the majority of patients in the United Kingdom (UK) although some children had MSK symptoms for more than a year before diagnosis [5]. When evaluating care pathways to appropriate care in children with MSK symptoms in the UK, for the majority of patients three specialties were involved before the first paediatric rheumatologist assessment [4]. Most referrals were from general paediatrics (56%) and orthopaedics (24%) and it took six months to six years before the first assessment by a paediatric rheumatologist from the time of presentation [4]. Many patients were referred back-and-forth between primary and secondary care specialties [4].

Evidence suggests that poor paediatric MSK clinical skills of non-rheumatology specialist healthcare workers could be a contributing factor to explain the delay in the diagnosis of MSK conditions [6]. Among 349 trainees and experienced doctors, 21% and 53% had ‘no’ or ‘some’ self-confidence in paediatric MSK examinations respectively in the UK [6]. These findings were also mirrored among primary care physicians and non-rheumatology specialists in East Africa where many reported a lack of confidence in MSK examination (55%) and diagnosing (68%) specific rheumatic conditions [7]. There was no definite correlation between physicians' confidence in performing an MSK evaluation and sex, country of education, specialization, number of years in practice, or number of rheumatic cases they self-reported as having diagnosed [7].

Foster et al. developed and validated the paediatric gait, arms, legs, and spine (pGALS) screening tool to detect abnormal joints with the ultimate intention of increasing earlier detection of paediatric rheumatic conditions by health care professionals who are not rheumatologists [8].

There are few paediatric rheumatologists in sub-Saharan Africa [9] and the majority of medical practitioners have little or no confidence in MSK examination [6]. The pGALS tool provides a feasible and pragmatic solution to facilitate earlier detection of MSK conditions with ultimately timely and appropriate referral. pGALS has demonstrated excellent sensitivity in detecting MSK abnormalities in English, Turkish, and Mexican languages [8, 10, 11].

In Kenya, paediatric residents at the AKUH and Kenyatta National Hospital were trained on the pGALS screening tool [12]. Written anonymized questionnaires were completed before and post-training to assess the participant’s level of confidence in examining the MSK system [12]. A total of 16 residents were assessed and 94% of these residents reported more confidence in the MSK examination after the training [12]. The pGALS has been in use at AKUH in its English format, but no official Kiswahili version exists for its Kiswahili-speaking patients. The purpose of this study was to develop a Kiswahili version of pGALS and undertake a validation exercise to determine its sensitivity and specificity in detecting abnormal joints in children and to determine its acceptability among parents and guardians.

## Methods

This was a screening utility study done between April 2020 to February 2021.

### Translation process

The following three screening questions of the pGALS tool were translated:

1. Question 1: Do you (or does your child) have any pain or stiffness in your (their) joints, muscles or back?
  Je una uchungu au ugumu kwenye vifundo, misuli au mgongo?
  Je mtoto wako ana uchungu au ugumu kwenye vifundo, misuli au mgongo?
2. Question 2: Do you (or does your child) have any difficulty getting yourself (him/herself) dressed without any help?
  Je unapata ugumu unapovaa nguo bila usaidizi wowote?
  Je mtoto wako ana ugumu wowote anapovaa nguo bila usaidizi wowote?
3. Question 3: Do you (or does your child) have any difficulty walking to school?
  Je unapata ugumu wowote unapotembea kwenda shuleni?
  Je mtoto wako anapata ugumu wowote anapotembea kwenda shuleni?

**Fig. 1:**
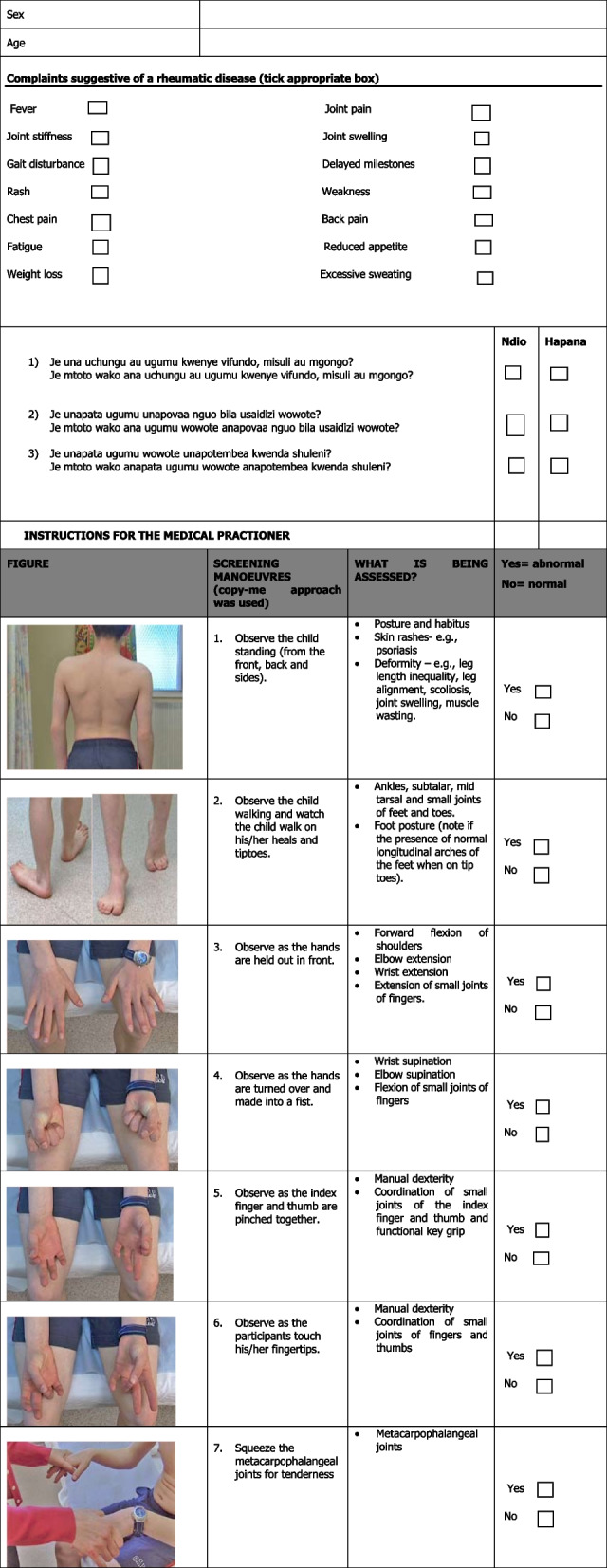
Kiswahili-pGALS

The translation was done according to the WHO standard for translation of a tool to achieve cross-cultural and conceptual equivalence. This was a six-step process [13]. Step 1 was forward translation of the screening questions from English to Kiswahili was done by two independent translators from ‘Global Languages Masters’ translation agency in Nairobi Kenya. In step 2, an expert committee consisting of the translators, the principal investigator, and the supervisors then met and agreed on an equivalence between the two translations. For step 3, back translation of the agreed-upon Kiswahili translated tool was done from Kiswahili to English by an independent translator from Global Languages Masters translation agency. In step 4, pretesting of the selected Kiswahili tool was then done on 25 children who spoke Kiswahili to determine any flaws in translation before the main study [14]. In step 5, modification of the Kiswahili tool was done by the expert team based on recommendations from the pre-test to produce the final version of the Kiswahili-pGALS screening tool. The Kiswahili- pGALS was then allowed to proceed for use in step 6 (Fig. 1).

### Design and study setting

This was a screening utility study conducted at AKUH in Nairobi, Kenya. AKUH is a 254-bed long term care facility offering general medical services, specialist clinics and diagnostic services. It is a premier, tertiary, teaching, and referral health care facility in sub-Saharan Africa.

### Study population and inclusion criteria

Participants were recruited at the accident and emergency department at AKUH based on their ability to speak Kiswahili and the presence of MSK and systemic complaints suggestive of MSK disease as per a review of the pGALS “pGALS – a screening examination of the musculoskeletal system in school-aged children” by Helen foster and S Jandial [15]. These complaints included fever, joint pain, joint stiffness, joint swelling, gait disturbance, delayed milestones, rash, weakness, chest pain, back pain, fatigue, reduced appetite, weight loss, and excessive sweating [15]. The ages 5–16 years were selected as they reflect school-aged children, similar to participants enrolled in the original pGALS study. Upon recruitment, the participants were triaged by a nurse and those who met the level V Paediatric Canadian triage and acuity scale (classified as non-urgent, to be seen by the doctor within 2 h) were selected by the research assistants as they were deemed medically stable.

The exclusion criterion was any patient already enrolled in the AKUH paediatric rheumatology clinic.

### Data collection and measures

The participants were recruited using systematic random sampling where every second patient who met the inclusion criteria was assessed on specific days of the week in line with the paediatric rheumatologist’s availability so they can have the Kiswahili-pGALS administered and the gold standard review on the same day.

Recruitment and consent were done by two research assistants (resident doctors) already pre-trained on the pGALS by a paediatric rheumatologist. Research assistants were chosen based on their fluency in Kiswahili, successful completion of an online pGALS training module supervised by the paediatric rheumatologist. Training was done via video presentation available at the ‘Paediatric Musculoskeletal Matters’ official website issued by Newcastle University and a certificate was issued upon successful completion [16].

One research assistant posed the screening questions (to either the parent/guardian or the child) and supervisedeach child as he/she performed the manoeuvres as per the pGALS screening tool using a “copy me” approach. The other research assistant observed the facial expressions of each child as the manoeuvres were being done, recorded these expressions (using a visual analogue scale), and administered questionnaires to the guardians or parents of the children. The questionnaires required demographic details of the guardians inclusive of level of education as well as Likert items about their opinion on level of discomfort their children experienced during the Kiswahili-pGALS and their opinion on the time it took. Guardians or parents were present during these procedures and acted as liaison when needed. The time taken to perform the Kiswahili-pGALS was recorded for each child. The outcome of the Kiswahili-pGALS was either a ‘positive’ or ‘negative’ result for MSK system abnormality. The Kiswahili-pGALS was considered positive if any of the screening questions or manoeuvres were positive for abnormal findings.

After being subjected to the Kiswahili-pGALS, each child was assessed on the same day by a paediatric rheumatologist. These children were classified as having normal or abnormal joints according to standard rheumatology practice (look, feel, move method of general MSK examination [17]. Any child who met the standard criteria of having abnormal joints was offered to follow-up at the paediatric rheumatology clinic.

### Study measures

Positive pGALS: The Kiswahili-pGALS was considered positive if any of the screening questions and manoeuvres were positive (or had ‘yes’ ticked).

Negative pGALS: The Kiswahili-pGALS was considered negative if all the questions and manoeuvres were negative (or had ‘No’ ticked).

Gold standard: This was the Paediatric Rheumatologist using standard rheumatology criteria for discerning abnormal joints (look, feel, move method).

## Pilot-test results

Following the translation of the tool in Kiswahili, we piloted the Kiswahili-pGALS on the first 25 patients recruited in the study. Thirty-six percent of the participants did not understand the word ‘vifundo’ (joint) whereas 32% could not understand the word ‘misuli’ (muscle) in screening question one after pGALS was translated into Kiswahili. The need for post hoc analysis was discussed following the difference in understanding the two terminologies and it was suspected that this was dependent on who responded to the screening questions between the parent/guardian or the children. It was concluded that although the words ‘vifundo’ and ‘misuli’ were difficult to understand, they were the standard Kiswahili translations of the respective anatomical part of the body and hence could not be substituted. A hypothesis was made that most children in the current Kenyan educational system are not taught these complex anatomical parts in Kiswahili before grade six (age group of 11–13 years). As per the pilot study, this age group made up only 20% of the sampled population. Children under the age of 11 years comprised 76% of the pilot study population and this could explain the percentage of participants that did not understand the two words. Therefore, parents were allowed to answer the screening questions on their child’s behalf in the study.

Eighty-eight percent of the participants considered the Kiswahili-pGALS acceptable at this stage and the mean time taken to complete it was just over five minutes. The Kiswahili-pGALS was found to be reliable based on a Cronbach’s alpha of 0.71 which is above the standard reliability cut off of 0.7.

A total of 100 patients were recruited for this study (pilot data included). This was due to recruitment challenges with lower patient numbers than anticipated in the ambulatory service following the COVID-19 pandemic and lockdown imposed in Kenya during the data collection period.

### Data analysis

Statistical analyses were performed using Stata 13. Demographic details were summarized using descriptive statistics. The sensitivity and specificity of the Kiswahili-pGALS tool were estimated from 2 × 2 tables (Table 1).

**Table 1:** 2 by 2 table of the Kiswahili p-GALS versus the gold standard diagnosis

pGALS outcome (Test)	Gold Standard (Condition)		
	**Abnormal**	**Normal**	**Total**
**Positive**	43 (TP)(a)	21 (FP)(b)	64
**Negative**	13 (FN) (c)	14 (TN) (d)	27
**Total**	56	35	91

*TP* True positive, *FP* False positive, *FN* False-negative, *TN* True negative

The sensitivity of the Kiswahili-pGALS was derived from the formula: Sn = a /(a + c). The specificity was derived using the formula: Sp = d/(b + d).

The positive and negative predictive values of the Kiswahili-pGALS were calculated to determine the probability that the child had a definitive rheumatology condition using the formulas, a/(a + b) and d/(c + d) respectively.

The diagnostic accuracy of the pGALS tool was calculated to reflect the overall capacity of the Kiswahili-pGALS, in correctly classifying subjects among all subjects, derived from the formula: (a + d)/n. AUC estimate with the corresponding 95^th^ confidence interval was derived from the ROC curve plotting the True Positive Rate (sensitivity) against 1-False Positive Rate (1-specificity) along various performance thresholds.

Post hoc analysis: Based on expert opinions after the piloting of the Kiswahili-pGALS screening tool, it was important to perform the following post-hoc analyses: a) Pearson’s chi-square test or Fisher’s exact test to assess the association between acceptability of the pGALS screening tool and respondents’ characteristics and b) to assess the differences in specificity and sensitivity of the tool between the respondents to the screening questions (parents and the child). As per the original pGALS tool, either the parent or the child could answer the screening questions and hence it was hypothesized that the specificity and sensitivity of the tool will be higher when parents responded than when the child responded.

## Study results

**Fig. 2:**
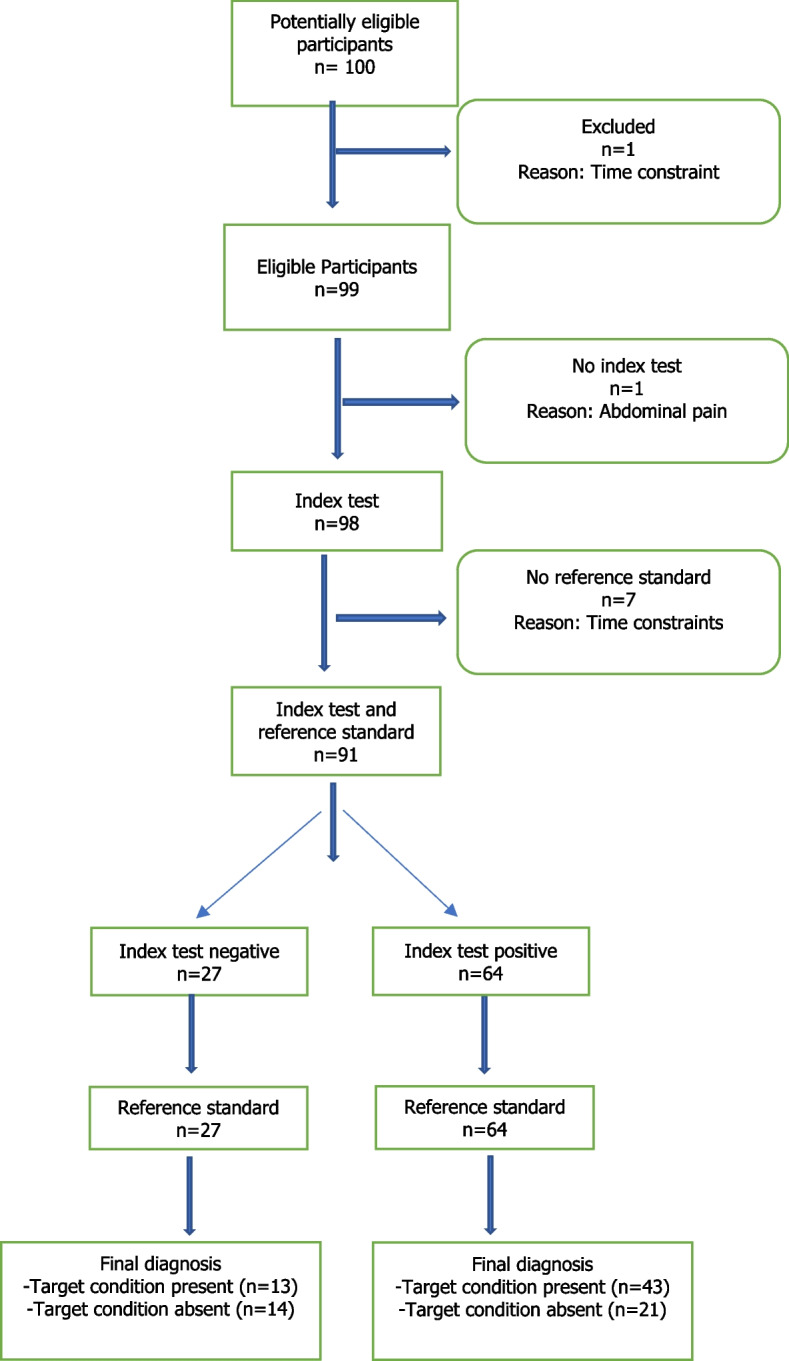
Participant's flow chart

The study recruited 100 participants where 91 children completed both phases of the study. The response rate was thus 91% (Fig. 2). The median age of the children enrolled in the study was 9 years (IQR 7–11) and majority (56%) were boys (Table 2). The median age of the accompanying caregiver was 39 years (IQR 34–44), majority (67.4%) of whom were female.

**Table 2:** Social demographic characteristics

Variables (*n* = 100)	n (%)
**Child’s gender (*n* = 100)**	
Male	54 (54)
Female	46 (46)
**Child’s age yrs. (median (IQR)**	9 (7–11)
**Parent age yrs. (median (IQR)**	39 (34–44)
**Parents’ gender (*n* = 98)**	
Male	32 (32.6)
Female	66 (67.4)
**Parents/guardian’s educational level (*n* = 98)**	
Primary	6 (6.1)
Secondary	20 (20.4)
Tertiary	72 (73.4)

The prevalence of rheumatic conditions in children coming to AKUN with any MSK symptom suggestive of a rheumatic disease was 61.5% (95% CI 51.0 to 71.1) (Table 3). The most common symptoms were a rash (76%), joint pain (29%) and reduced appetite (17%). The main rheumatic variants identified by the gold standard were pes planus (82.1%), joint hypermobility (61.5%), and calcaneus valgus (8.9%).

**Table 3:** Distribution of the Kiswahili-pGALS parameters

Number of positive Kiswahili-pGALS manoeuvres in the participants (*n* = 98)	
0	35 (35.7)
1	44 (44.9)
2	11 (11.2)
3	5 (5.1)
≥ 4	3 (3.0)
**Kiswahili-pGALS outcome (*n* = 98)**	
Positive	67 (68.0)
Negative	31 (32)
**Gold standard's diagnosis (*n* = 91)**	
Normal joints	35 (38.5)
Abnormal joints	56 (61.5)
**Spectrum of conditions (*n* = 56) (Categories were not mutually exclusive)**	
Pes planus	46 (82.1)
Joint hypermobility	23 (41.1)
Calcaneus valgus	5 (8.9)
Tight muscles	2 (3.6)
Osgood Schlatter disease	1 (1.8)
Soft tissue injury	1 (1.8)
Ankle pain	1 (1.8)
Knee pain	1 (1.8)
Antalgic gait	1 (1.8)
Left-sided hemiparesis	1 (1.8)
Genu Varus	1 (1.8)
**Level of discomfort of participants as perceived by the parent/guardians (*n* = 98)**	n (%)
No discomfort	83 (84.7)
Mild pain	6 (6.1)
Moderate pain	3 (3.1)
Lots of pain	1 (1.0)
Some discomfort	5 (5.1)
**Level of discomfort of participants as perceived by the research assistant (*n* = 98) (Visual analogue scale)**	
No hurt	86 (87.8)
Hurts a little	8 (8.2)
Hurts even more	2 (2.04)
Hurts a whole lot	2 (2.04)

*IQR* interquartile range, *SD* standard deviation

Among the 99 participants who responded to the screening questions, 29 (30%) had pain or stiffness in their joints, muscles, or back, 3 (3.0%) had difficulty dressing without help and 9 (9.1%) had difficulty walking to school.

Thirty-five (35.7%) of the 98 children who completed the Kiswahili-pGALS were able to perform all the manoeuvres optimally while 44 (44.9%) could not perform at least one manoeuvre. Most of the screened children could not perform manoeuvre two (55.6%), manoeuvre 14 (11.2%) and manoeuvre one (9.1%) optimally. All the children performed the eleventh and twelfth manoeuvre.

Sixty-seven of the 98 participants (68%) had a positive Kiswahili-pGALS. It took a median of 5.0 min (IQR 3.5–6.0) to complete the Kiswahili-pGALS. It took longer to complete the pGALS screening tool for children with a positive outcome (median: 5.1 min, IQR 3.5–6.0) compared to those with a negative outcome (median: 4.2 min, IQR 3.5–5.3), though the difference was not statistically significant (*p* = 0.120).

A post-hoc analysis showed that there was no association between the outcome of the screening test (pGALS findings) and the person (either child or parent) who responded to the screening questions (*p* = 0.637), level of discomfort (*p* = 0.242), and time taken to complete the screening process (*p* = 0.103).

The Kiswahili-pGALS screening tool had a screening sensitivity (*true-positive rate*) of 76.8% (43/56; 63.6–87.0%) and a screening specificity (*true-negative rate*) of 40.0% (14/35; 23.9–57.9%). The screening tool had a miss rate (*false negative rate*) of 23.2% (*n* = 13/56) and a false alarm rate (*false positive rate*) of 60% (*n* = 21/35).

**Fig. 3:**
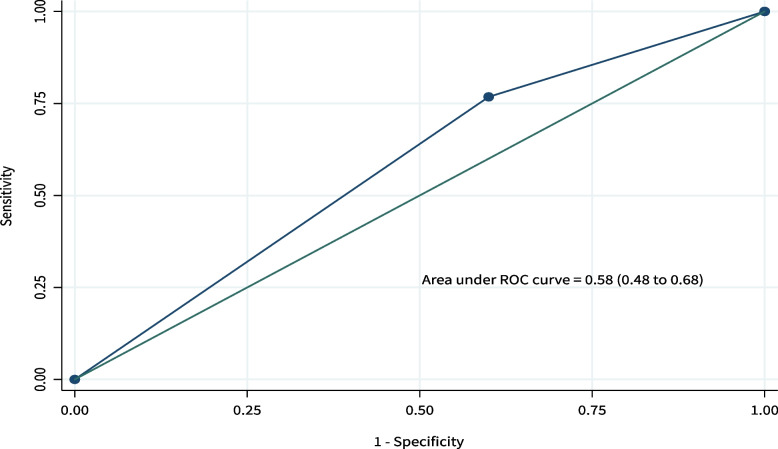
Receiver operating characteristics curve

The area under the receiver operating characteristic curve was 0.58 (0.48–0.71 (Fig. 3).

Most of the respondents (84.7%, *n* = 83/98) reported no discomfort during the screening process with only one (1%) complaining of pain while the research assistants reported no discomfort for 87.8% (*n* = 86/98) of the cases.

The acceptability rate of the Kiswahili-pGALS screening tool was 82.0% (*n* = 80/98; 73.1–88.4%).

## Discussion

The Kiswahili-pGALS had a sensitivity of 76.8% and a specificity of 40%. These were lower statistics than observed in previous validation studies, however, the demographics of the participants and the level of acceptability of the screening tool by parents/ guardians were similar.

Amongst Mexican and Turkish children, the tool’s sensitivity was 97% [11] and 93.7% [10] respectively. The original pGALS validation study in the UK also observed a sensitivity of 97–100% [8]. It is likely that due to our study centre (private, single-centre, referral institution), the study population was one of middle-high income and that the participants recruited may have still been in a much earlier phase of the disease. For example, in the study by Ezgi et al. among Turkish children, most of the participants that tested positive on the pGALS (14/17 participants) were recruited from the outpatient clinic of Physical Medicine and Rehabilitation (PM&R) [10]. The Turkish study described the outpatient clinic as a centre having patients with ‘more chronic and MSK problems’ [10]. This may have led to selection bias for a validation study as most of the participants had chronic joint problems which are major symptoms of musculoskeletal disease. The difference between the prevalence of MSK conditions at two points of recruitment in the Turkish study; the emergency department vs the PM&R outpatient clinic was statistically significant (*p* = 0.003) [10] hence the higher sensitivity of the Turkish-pGALS.

It is also important to point out the differences in methodology used in previous pGALS validation studies. In the cross-cultural validation of the pGALS among Mexican children, children already diagnosed with rheumatic diseases attending outpatient clinics were recruited and divided into 3 groups [11]. Although the research assistant was blinded to the diagnosis of the participants, this methodology could be a source of selection bias and may contribute to the differences in the diagnostic utility of the pGALS between the Mexican study and the Kiswahili-pGALS study. These children would have been more likely to accurately respond to the screening questions as they may have been subjected to the test in the past or have better recall of the investigated symptoms. They may even have been more likely to perform the manoeuvres better as opposed to the methodological approach taken in the Kiswahili-pGALS leading to a seemingly higher sensitivity of 97% and a specificity of 93% among the Mexican children [11]. In the Turkish-translated pGALS study, children were recruited from the paediatric emergency department and physical medicine and rehabilitation clinic and subjected to the pGALS [10]. Only those who were positive for the pGALS were assessed using the gold standard which was an invalid approach in methodology as there was no comparison group at the level of the gold standard. The Kiswahili-pGALS study translation was robust following appropriate stages of WHO translation and is less likely to have suffered the shortfalls inherent in the other studies.

The specificity of the Kiswahili-pGALS screening tool is the lowest seen among other translated pGALS tools (24–26), which is likely to be due to the differences in methodologies in previous studies as described above. It is also important to note that pGALS is not a screening test used to make a diagnosis but instead to flag up joints that are not regarded as normal. In addition, a higher false positive rate (60%) is preferable to a higher false negative rate (which was as low as 23% in our study) as the false positives would still be referred to a paediatric rheumatologist while missed false negative cases would lead to unchecked disease progression and poor outcomes. The Kiswahili- pGALS therefore, which may still be useful in aiding early detection, although a relatively large number of false positives would still need to be reviewed by a rheumatologist.

Overall, the Kiswahili-pGALS also noted more mechanical conditions than inflammatory conditions and this may have also influenced the findings. The most common symptom exhibited by the children screened was rash (76%) which did not align with the most common rheumatic variant (Pes planus). However, of the 98 children who completed the Kiswahili-pGALS screening, only 9.1% had challenges with manoeuvre one which would have been influenced by the presence of rashes. On the other hand, 55.6% of the children screened had trouble with manoeuvre two explaining why eventually mechanical variants like pes planus were the most common rheumatic conditions diagnosed by the gold standard. It is therefore unlikely that using the presence of rashes as an inclusion criterion may have influenced the diagnostic accuracy of the screening tool.

The overall acceptability of the Kiswahili-pGALS screening tool was lower than 93–98% observed in other pGALS studies [8, 10, 18–20]. However, this tool was considered acceptable to any parent who deemed the time taken to be ‘adequate’ or ‘little’ and found that their children experienced no discomfort during the study. This was a different approach from other studies where a visual analogue scale (smiley faces) was provided to the parents and they were requested to decide whether the tool was simply acceptable [18, 20]. Most guardians felt their children experienced no discomfort during the administration of the Kiswahili-pGALS screening tool. Their assessment of the level of discomfort was not associated with their gender, education level, or length of waiting time before the Kiswahili-pGALS screening. There was no agreement between the parents’/guardians’ opinions and research assistants’ assessment of comfort via the visual analogue scale and this has potential for further research in the future.

It took slightly longer to complete the Kiswahili-pGALS tool (5.0 min) in comparison to other pGALS translation studies such as the 4.4 min in Peru [20] and four minutes in Malawi and Turkey [10, 18]. It was even longer than the 2–3 min taken to complete the tool in Mexican and British studies [8, 11, 19]. The length of time may have been contributed by the time it took to answer the three screening questions in the Kiswahili-pGALS probably due to the complexity of the anatomical parts in question, but further analysis was however beyond the scope of the study and was not assessed.

Post hoc analysis showed that when children responded to the screening questions in the pGALS, the time taken was slightly longer although this difference was statistically insignificant. It also took longer to complete the Kiswahili-pGALS in children who ended up with a true rheumatic condition than those with a negative outcome although this difference was also statistically insignificant. This pattern was similarly seen in the Turkish-pGALS where the difference in time taken to complete the pGALS in participants eventually diagnosed with a rheumatic disease was not statistically significant (*p* = 0.10) [10]. Despite the length of time, many of the guardians found the time to complete the Kiswahili-pGALS acceptable.

## Conclusion and recommendations

Further studies with more diverse cohorts of participants and multiple gold standards in different study centres are recommended for the results to be generalizable and to assess interrater reliability.

The Kiswahili-pGALS had a lower sensitivity and specificity than other studies. Nonetheless, the sensitivity is still within acceptable range for a screening tool.

### Limitations

This study was done in a single centre and therefore may not be a complete representation of the Kenyan population and hence results cannot be generalised. The was also just one gold standard, although acceptable we would have had better quality of results with multiple gold standards. Of note the study was done during the Covid 19 pandemic limiting our sample size even further.

## Data Availability

Data extraction files are available upon reasonable request.

## Abbreviations

AKUH: Aga Khan University Hospital
CI: Confidence Interval
GALS: Gait, arms, legs, and spine tool
MSK: Musculoskeletal
N: Sample size
pGALS: Paediatric gait, arms, legs, and spine tool
P: Prevalence
ROC: Receiver operating curve
Sn: Expected sensitivity
UK: United Kingdom
W: Assumed 95% confidence width
WHO: World health organisation
